# Comparison of proinsulin and C-peptide secretion in healthy versus long-standing type 1 diabetes mellitus cohorts: A pilot study

**DOI:** 10.1371/journal.pone.0207065

**Published:** 2018-11-09

**Authors:** Catherine A. Sullivan, Jose M. Cacicedo, Iniya Rajendran, Devin W. Steenkamp

**Affiliations:** 1 Section of Endocrinology, Diabetes and Nutrition, Boston University School of Medicine, Boston, MA, United States of America; 2 School of Public Health, Boston University, Boston, MA, United States of America; La Jolla Institute for Allergy and Immunology, UNITED STATES

## Abstract

**Aims:**

Increased proinsulin (PI) compared to C-peptide (CP) concentrations have been reported, both prior to type 1 diabetes mellitus (T1D) onset, as well as early in disease. In this pilot study, we sought to define the normal PI secretion in a healthy cohort and compare this to a local T1D cohort and a separate well-defined nationally representative T1D cohort with measurable CP.

**Methods:**

Thirteen healthy subjects and 12 T1D subjects with T1D >3 years from the local T1D cohort completed mixed meal tolerance tests (MMTT) with PI and CP measured over 90 and 240 minutes. The change in CP (maximum versus baseline, ΔCP) during MMTT in the T1D Exchange T1D cohort was stratified according to non-fasting PI concentrations, based on a fasting PI threshold, as defined by the healthy control group.

**Results:**

The maximum fasting PI in the control group was 6 pmol/L. Individuals from the T1D Exchange with a non-fasting PI ≥ 6 pmol/L had a lower ΔCP during a MMTT, compared to those with a PI < 6 pmol/L. While only three individuals from the local T1D cohort had measurable CP and PI during the MMTT, those with a greater ΔCP had lower PI secretion.

**Conclusion:**

While all T1D subjects from the T1D Exchange secreted measurable non-fasting PI, those with a greater non-fasting PI demonstrated a decrease in ΔCP during the MMTT. PI may be preferentially secreted compared to CP in some individuals with long standing T1D.

## Introduction

Type 1 diabetes mellitus (T1D) is characterized by T-cell-mediated autoimmune destruction of insulin secreting pancreatic beta cells. This process precedes the diagnosis of overt hyperglycemia in most individuals, and ultimately progresses to result in near-absolute beta-cell failure, leading to life-long exogenous insulin dependence [1]. Recently, the long standing dogma that beta cells do not function in T1D has been challenged as endogenous insulin secretion measured by C-peptide (CP) appears to persist for decades in many individuals with long standing TID [2–5]. Measurement of serum CP concentrations during a standardized mixed meal tolerance test (MMTT) is a well-established, practical method of assessing nutrient stimulated beta cell secretory function [6], with peak CP concentrations > 0.2 nmol/L being associated with improved glycemic control, less hypoglycemia and fewer microvascular complications in individuals living with T1D [7,8].

Normal insulin biosynthesis is a multi-step process, beginning with a pre-prohormone, preproinsulin [9]. Preproinsulin is synthesized in the rough endoplasmic reticulum of the beta cell and translocated to the cytosol of the rough endoplasmic reticulum where it is converted to proinsulin (PI). Proinsulin is then transported to the Golgi apparatus to become incorporated into a new “immature” beta-granule where it is subsequently cleaved into equimolar amounts of insulin and CP via prohormone convertases (PCSK1, PCSK2, and carboxypeptidase E), marking the transformation to a mature beta-granule, ready for exocytosis in response to various stimuli.

While not typically measured as a clinically useful marker of beta cell secretory function, serum PI has been reported to be elevated in relation to CP (increased PI:CP ratio) in individuals who are at increased risk for development of T1D, as well as soon after diagnosis [10,11]. In healthy rodent islets, some PI is secreted along with CP and insulin when beta cells are stimulated [12]. In addition, islets of db/db mice have increased PI synthesis rates and increased PI secretion under basal glucose concentrations. However, insulin secretion is abnormal, suggesting the PI may be preferentially or prematurely released in certain situations [13]. Elevated serum PI:CP may be a marker of beta cell endoplasmic reticulum dysfunction and supports the notion of residual dysfunctional “stressed” beta cells in individuals with T1D [11]. Given that CP is cleaved from PI, it may not be surprising that PI would be measurable in individuals with measurable CP, especially if there is inefficient beta cell processing [14]. Recently, we and others, have reported that PI is measurable in sera and pancreatic extracts in individuals with long standing T1D despite unmeasurable CP [15–17]. The biological relevance and possible mechanisms that explain this finding remain to be elucidated and multiple questions remain unanswered. In this pilot study we therefore sought to answer the following questions: How does PI secretion during a MMTT compare in long standing T1D to healthy individuals? Could further evaluation of PI secretion from a larger nationally representative T1D cohort further our understanding of normative PI prevalence data in the T1D population?

### Subjects, materials and methods

All research activity was performed at Boston Medical Center in Boston, MA. For the healthy and local type 1 diabetes cohort, this study was approved by the Boston University IRB, IRB Number H-35298. Written consent was obtained from subjects. Consent was not obtained from the data from the T1D Exchange cohort as the data were analyzed anonymously.

### Healthy cohort

Thirteen healthy volunteers between the ages of 18 and 65 who had no history of diabetes, impaired glucose tolerance or risk factors for diabetes as well as no family history of diabetes in a first-degree relative were eligible for participation. They underwent two consecutive standardized mixed meal tolerance tests (MMTT) at least one week apart. Subjects were excluded if their fasting blood glucose was ≥ 5.6 mmol/L prior to the MMTT.

After an overnight fast, 90-minute (4-point) and subsequently a 240-minute (11-point) MMTT were completed to ascertain the typical profiles of CP, PI and glucose in response to a nutrient stimulus. Each subject consumed a liquid meal consisting of Ensure Plus (Abbott Nutrition) at 6cc/kg (max 360 cc), content per 100 ml: carbohydrate 21.5 grams, protein 5.5 grams, fat 4.6 grams, energy 147 kcal. Whole blood capillary glucose was measured via a calibrated glucometer and whole blood collected in red-capped BD vacutainers via an indwelling catheter at each of the 4 (time 0, 15, 30, and 90 minutes) and 11 time points (time 0, 5, 15, 30, 60, 90, 120, 150, 180, 210 and 240 minutes). Whole blood samples were allowed to clot at room temperature for 30 minutes after which they were centrifuged at 1,200 x g for 15 min and serum frozen and stored at -80 degrees Celsius until assayed. PI and CP were measured from serum following a single thaw after the study visit. The initial 90-minute MMTT and first 90-minute analyses of the subsequent 240-minute MMTT data for each individual subject was compared to ensure reproducibility of the protocol.

### Type 1 diabetes mellitus cohorts

Twelve individuals with established T1D were recruited from the Diabetes Clinic at Boston Medical Center and underwent a single 240-minute (11-point) MMTT with identical measurements of CP, PI and glucose, as described above. These individuals were aged between 18 and 65, had a BMI between 18 and 30 kg/m^2^ and a clinical diagnosis of T1D. T1D was defined by the presence of detectable islet specific auto-antibodies in the serum and/or a history of full dependence on insulin within 6 months of diagnosis. Only those with disease duration of >3 years and who were dependent on exogenous insulin were eligible for participation. Presence or absence of measurable CP was not an inclusion criterion for this cohort. Subjects on multiple daily injections of insulin were instructed to inject their basal (long acting) insulin the evening prior to the study visit and to omit all rapid acting insulin within 2 hours of the start of the study visit. Basal insulin typically injected in the morning was held until after the MMTT was completed. Subjects using continuous subcutaneous insulin infusions were instructed to continue their usual basal insulin infusion rates and also avoid bolus or correction doses of insulin within 2 hours of their study visit. All subjects were required to have a capillary blood glucose value between 3.9 mmol/L and 8.4 mmol/L prior to the start of the MMTT.

Non-fasting frozen sera from 100 individuals with well-characterized T1D were obtained from the T1D Exchange Clinic Network participants who were part of the Residual CP study [3,18]. At initial study entry, non-fasting blood samples were obtained, with participants instructed to eat within 4 hours prior to their baseline blood draw. Insulin was administered as per usual routine. Meal carbohydrate intake and time of meal were recorded. Individuals with measurable CP at study entry (>17 pmol/L) then completed a MMTT visit within 35 days, with time points measured at -10, 0, 30, 60, 90, and 120 minutes, as previously described [3]. In addition to the non-fasting samples, baseline and peak MMTT-stimulated CP concentrations for each subject were provided by the Residual CP Study team to include in our analysis. We used the baseline non-fasting samples obtained at study entry to measure non-fasting PI concentrations.

As our goal was to measure beta cell secretory products in a well-defined long-standing T1D population, subjects were excluded from the T1D Exchange cohort analysis if they were ≤ 3 years from initial diagnosis. In this cohort we additionally excluded subjects who did not have a measurable CP at time of study entry (in order to ensure we would be able to measure the precursor hormone, PI, in relation to CP concentrations), those who were hypoglycemic at the time of blood collection (defined as serum glucose <3.9 mmol/L) or if the time of collection was less than 15 minutes post meal (to ensure sample collection was non-fasting). As we previously described in this cohort, 16% of the CP negative individuals had measurable non-fasting PI concentrations, albeit in low concentrations [17]. Based on these criteria, a total of 37 T1D subjects from the T1D Exchange were included in this analysis.

### Laboratory measurements

#### Glucose

Whole blood capillary glucose measurements during the MMTT were measured on the Precision Xtra glucometer (Abbott). Serum glucose concentrations were determined with the Glucose (GO) Assay Kit (Cat. No. GAGO20- 1KT) from Sigma (St. Louis, MO) that employs the glucose oxidase and peroxidase method. All samples were diluted 1:10 with distilled water prior to measurement. Glucose concentrations from the T1D Exchange cohort were provided as previously described [3].

#### C-peptide and Proinsulin

CP concentrations for both MMTT cohorts and the non-fasting T1D Exchange samples were measured using the Mercodia (Winston Salem, NC) Ultrasensitive CP ELISA (Cat. No. 10-1141-01) which has a detection limit < 2.5 pmol/L and no cross-reactivity to insulin, but does have 5% cross reactivity to PI. Our measured intra-assay CV was 8.6% and inter-assay CV was 9.9% for this kit. Baseline and peak CP concentrations from the T1D Exchange MMTT were measured by the T1D Exchange Biobank, as previously described [3].

PI was measured using the ultrasensitive STELLUX Chemi Human Total Proinsulin ELISA (Cat. No.80-PINHUT-CH01, ALPCO, NH; using the updated ALPCO standard, post April 2017) that has a detection limit 0.14 pmol/L. In our experience intra-assay CV was 5.9% and inter-assay CV was 2.4%. As per the manufacturer, the assay fully measures intact PI and the Des (31,32), and Des (64,65) isomers of PI, with <0.6% cross reactivity to human insulin, 0% to human CP, and 0% to human glucagon. We have independently confirmed and validated the accuracy of this PI kit as previously described [17].

### Statistics

Variables with a skewed distribution were expressed as median with interquartile ranges (IQR); those with a normal distribution were expressed as mean ± SD or SE where specified. Statistical comparisons for non-parametric data between two groups were done by Mann-Whitney *U* test, except when data was categorical, in which instance a Fisher’s exact test was performed. All statistical analyses were performed using GraphPad Prism version 5.01 for Windows (GraphPad Software, San Diego, CA). P-values are two sided with a 0.05 significance level.

## Results

### Healthy cohort

**Fig 1:**
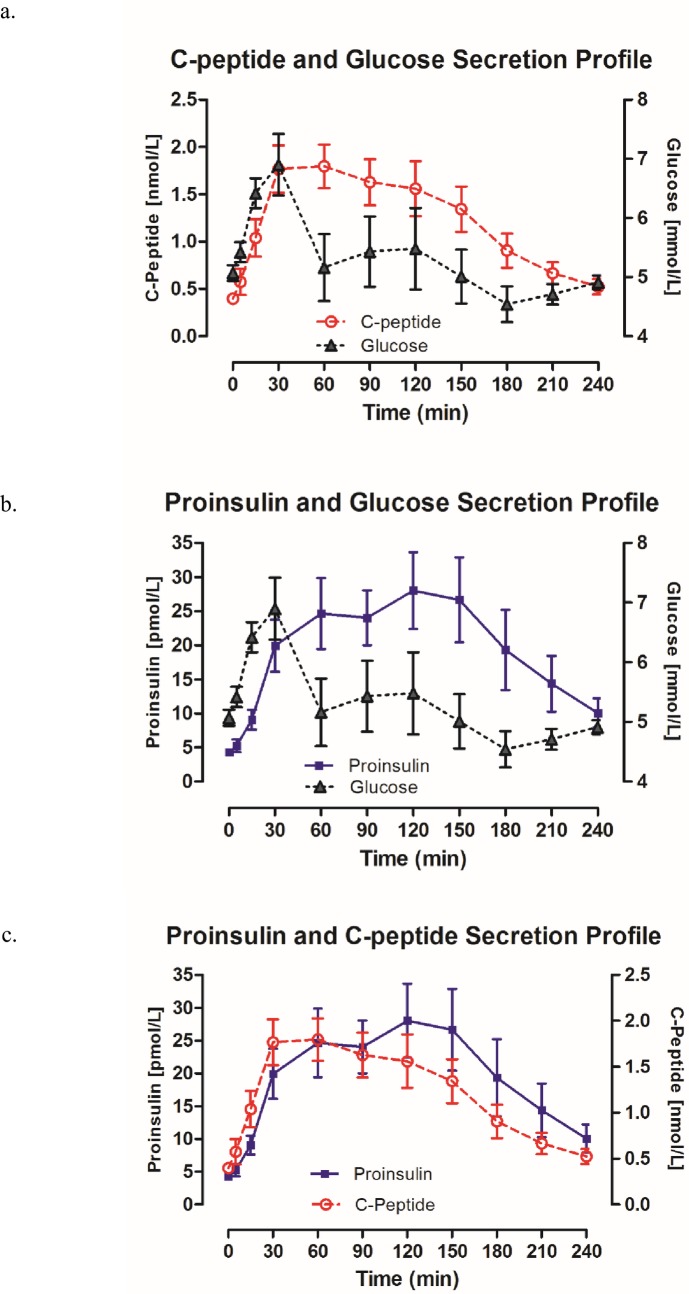
Secretion profiles of healthy cohort (n = 13) following a standard MMTT. Values measured at 11 time points over a 240-minute collection period. a. C-peptide and glucose secretion b. Proinsulin and glucose secretion c. Proinsulin and C-peptide secretion.

**Fig 2:**
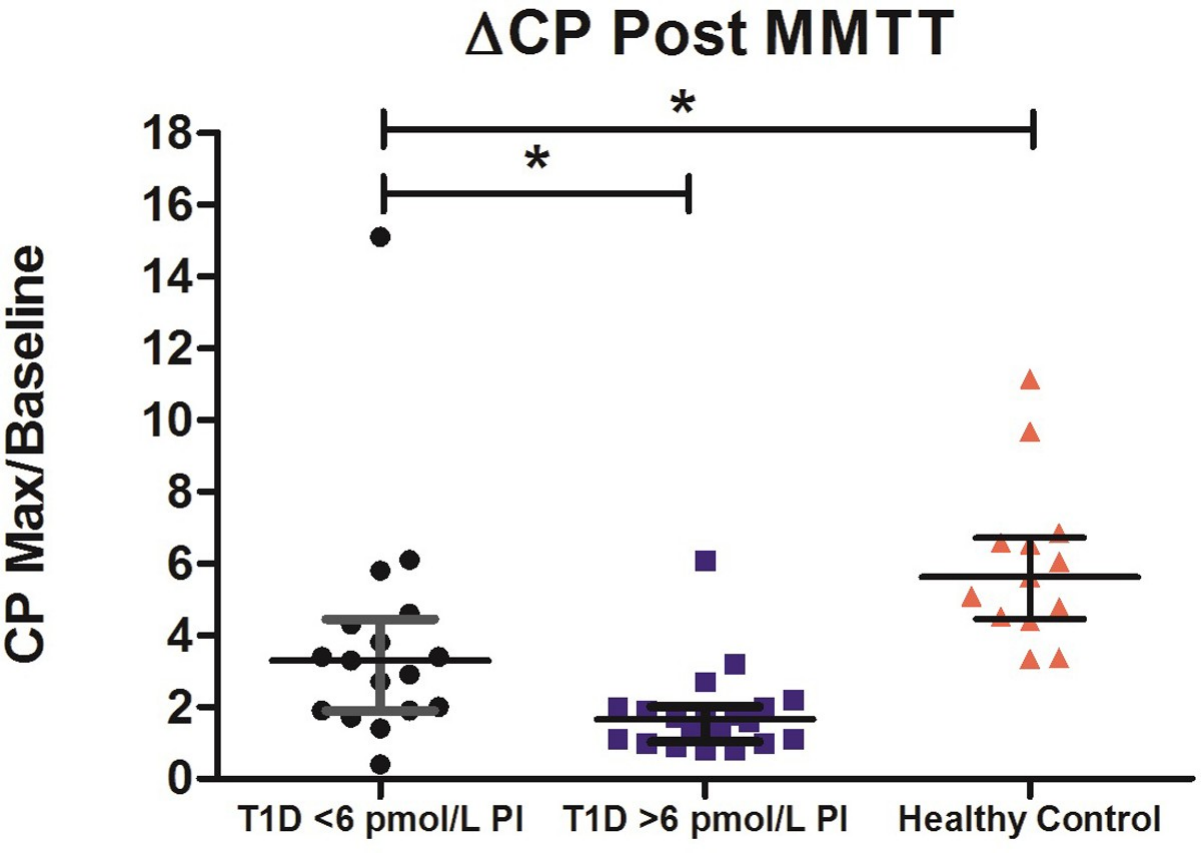
Change in CP (median with IQR) during MMTT, stratified by non-fasting PI (T1D) versus controls. Change in CP in the healthy cohort was greater than either T1D cohort. Change in CP in the T1D group with a non-fasting PI below 6 pmol/L was 2 times greater than those with a non-fasting PI above 6 pmol/L, suggesting differences in nutrient stimulated beta cell response. Data passed the Kruskal-Wallis test (p <0.0001) with Dunn’s post-test used to compare groups. *P-value ≤ 0.05.

The 13 individuals in the healthy control group (6 male and 7 female) were 27 ± 4.4 years and had a BMI of 22.3 ± 2.0 kg/m^2^. CP, glucose and PI were measured at all 4 and subsequent 11-time points. As data obtained from each individual subject’s 90 and 240-minute MMTT’s were highly correlated, we present only the full 240-minute MMTT data here in Table 1 and Fig 1A–1C. Both PI and CP followed a similar excursion profile, with PI peaking at a later time as compared to CP. The minimum fasting (baseline) PI was 2.39 pmol/L, with a mean of 4.3 pmol/L ± 1.9. We defined a “stimulated” or non-fasting PI concentration in our T1D cohort based on the maximum mean fasting value in the healthy cohort for the purposes of our comparative analysis. A PI concentration > 6 pmol/L was therefore considered to be a “stimulated” PI concentration in the T1D cohort. The median change in CP, (ΔCP), an index of nutrient stimulated β-cell function calculated by dividing maximum CP by baseline CP during MMTT, for the healthy cohort was 5.62 (4.44, 6.71) (Fig 2 and Tables 1 and S1 and S2). The peak PI:CP ratio during MMTT in the healthy cohort was 1.37 (0.87, 2.39).

**Table 1:** Healthy control group and local T1D cohort characteristics and MMTT results.

Characteristics	Healthy Cohort	Local T1D Cohort
**Number participants**	13	12
**Age (years)** ^**a**^	27 ± 4.4	37 ±12
**Female**	7 (54%)	2 (16%)
**BMI (kg/m**^**2**^**)** ^**a**^	22.3 ± 2.0	22.8 ± 1.8
**Age of Diagnosis (years)**	n/a	16 (12, 22.8)
**Duration TID (years)**	n/a	16 (12, 26)
**HbA1C (mmol/mol)**	n/a	54 (48, 70) 7.1% (6.5, 8.6)
**Baseline PI (pmol/L)**	3.38 (3.00, 5.31)	0.24 (0.19, 4.79)^b^
**Max PI (pmol/L)**	29.60 (18.50, 58.00)	2.95 (2.05, 9.78) ^b^
**Δ PI**	8.76 (6.17, 10.9)	8.54 (2.04, 15.50) ^b^
**Baseline CP (nmol/L)**	0.361 (0.275, 0.450)	0.003 (0.003, 0.680) ^b^
**Max CP (nmol/L)**	2.044 (1.479, 2.731)	0.910 (0.056, 0.120) ^b^
**Δ CP**	5.62 (4.44, 6.71)	21.5 (2.90, 35.3) ^b^
**Peak PI: CP (%)**	1.37 (0.87, 2.39)	3.60 (3.20, 4.90) ^b^

Change in PI or CP (ΔPI and ΔCP) defined as maximum over basal value during MMTT.

Values listed as medians with interquartile ranges in parenthesis.

a. Mean listed with SD

b. Results listed for 3 subjects with measurable CP and PI during MMTT

### Local T1D cohort

The 12 individuals in the local T1D group (10 male and 2 female) had a mean age of 37 ±12 years (range 20–59) and mean BMI of 22.8 ± 1.8 kg/m^2^ (Table 1.) The median age of diagnosis of T1D was 16 (12, 22.8) year, with median duration of T1D of 16 (12, 26) years. Average hemoglobin A1C (HbA1C) was 60 mmol/mol (7.6%.). Of the 12 subjects, only 3 had evidence of stimulation (increase from baseline) in both CP and PI during the MMTT (Tables 2 and S1 and S2), with a peak value of both CP or PI reached between 120–180 minutes. Subject 1 had a peak PI at 180 minutes, subject 2 at 120 minutes and subject 3 at 150 minutes. CP peaked at 90 minutes in subject 1, 180 minutes in subject 2 and at 150 minutes in subject 3. The peak PI:CP molar ratios (amount of proinsulin secreted as a percent of C-peptide secreted) for these 3 subjects were 3.6, 4.9 and 3.2%. Maximum PI during the MMTT in the 3 subjects was 2.05, 9.78 and 2.95 pmol/L with ΔCP of 21.5, 2.9 and 35.3 for subjects 1, 2 and 3, respectively. Three of the remaining 9 subjects did not have CP or PI detected during the MMTT, while the other 6 had very low CP at random time points (highest 14.4 pmol/L) without measurable PI. No subjects had measurable PI without having measurable CP.

**Table 2:** Local T1D cohort MMTT data: 3 Subjects with measurable proinsulin.

	Subject		
Characteristics	1	2	3
**Age (years)**	34	20	32
**Sex**	Male	Male	Female
**BMI (kg/m**^**2**^**)**	22.6	19.0	25.0
**Age of Diagnosis (years)**	18	15	28
**Duration TID (years)**	16	5	4
**HbA1C (mmol/mol)** **%**	72 (8.7)	98 (11.1)	55 (7.2)
**Baseline PI (pmol/L)**	0.24	4.79	0.19
**Max PI (pmol/L)**	2.05	9.78	2.95
**Δ PI**	8.54	2.04	15.5
**Basal CP (nmol/L)**	0.003	0.068	0.003
**Max CP (nmol/L)**	0.056	0.120	0.091
**Δ CP**	21.5	2.9	35.3
**Peak PI: CP (%)**	3.6	4.9	3.2

Change in PI or CP (ΔPI and ΔCP) defined as maximum (peak) over basal value during MMTT.

### T1D cohort, T1D Exchange (Table 3)

The thirty-seven included subjects from the T1D Exchange had measurable PI in their non-fasting samples with a median of 6.38 (2.67, 8.87) pmol/L and range of 0.66 to 74.52 pmol/L (S3 Table). Subjects were stratified into two groups based a non-fasting PI of 6.0 pmol/L, the cutoff derived from the healthy control group as described above. Seventeen (13 female and 4 male) subjects had a value <6 pmol/L and 20 (13 female and 7 male) >6 pmol/L. The median age for the <6 pmol/L group at time of study visit was older than the >6 pmol/L group: 48 (39, 54) and 38.5 (29.5, 43.5) years, respectively. However, there was no difference between the median age at diagnosis (29 (21, 39) versus 28.5 (22.3, 34.8) years)) or duration of T1D 9 (5.5, 22) versus 6 (5, 12.5)) years between the two groups. Further, they did not differ in glycemic control with HbA1C 55 (51, 64) versus 53 (41, 70) mmol/mol in the group with non-fasting PI <6 pmol/L and >6 pmol/L, respectively. There was no significant difference between the time of sample collection post meal, carbohydrate intake or glucose concentration at the time of collection.

**Table 3:** Non-fasting and MMTT results from T1D cohort stratified by non-fasting PI.

	PI < 6 pmol/L	PI > 6 pmol/L	*P* value^a^
**Subjects (n)**	17	20	
**Age (years)**	48 (39, 54)	38.5 (29.5, 43.5)	0.027
**Female (n)**	13 (76%)	13 (65%)	0.50^b^
**Age at Diagnosis (years)**	29 (21, 39)	28.5 (22.3, 34.8)	0.71
**Duration T1D (years)**	9 (5.5, 22)	6 (5, 12.5)	0.13
**HbA1C (mmol/mol)** **%**	55 (51, 64) 7.2 (6.8, 8)	53 (41, 70) 7.0 (5.9, 8.6)	0.40
**Non-Fasting, Post Meal Sample**			
**PI (pmol/L)**	2.62 (1.67, 4.17)	8.59 (7.25, 10.50)	<0.0001
**CP (nmol/L)**	0.035 (0.130, 0.052)	0.113 (0.064, 0.526)	0.0009
**PI:CP (%)**	7.8 (5.1, 15.7)	7.7 (4.1, 14.9)	0.99
**Carbohydrate intake** **(grams)**	30 (22, 40)	23.5 (17, 45)	0.60
**Time post meal (minutes)**	75 (38, 152.5)	95.5 (53.8, 131.3)	0.46
**Glucose (mmol/L)**	10.8 (9.3, 13)	13.6 (10, 20.5)	0.09
**Mixed Meal Tolerance Test**			
**ΔCP**	3.3 (1.9, 4.5)	1.65 (1.0, 2.0)	0.0025

Values listed as medians with interquartile ranges in parenthesis. Means listed with SE. Change CP (ΔCP) defined as maximum over baseline value during MMTT.

**a.** *P* value by Mann-Whitney *U* test,

**b.** Fishers test

Compared to those with a PI below the stimulated cutoff, those with a PI > 6 pmol/L had significantly greater non-fasting PI and CP concentrations with a median PI of 8.59 (7.25, 10.50) pmol/L versus 2.62 (1.67, 4.17) pmol/L and CP concentration of 0.113 (0.064, 0.526) nmol/L versus 0.035 (0.130, 0.052) nmol/L. As illustrated in Fig 2, the group with non-fasting PI >6 pmol/L had a lower ΔCP during their MMTT than those who had a non-fasting PI <6 pmol/L (1.65 (1.0, 2.0) versus 3.3 (1.0, 4.5)). There was no significant difference in the non-fasting PI:CP ratios between the two T1D Exchange groups.

## Discussion

Measuring PI and CP after MMTT is a practical and reliable means to assess nutrient stimulated beta cell function. To the best of our knowledge, this is the first time the profiles of both PI and CP have been determined in healthy and long standing T1D cohorts. In our local cohort, 3 of 12 of a T1D cohort had both measurable CP and PI during the MMTT. One subject had T1D for 18 years, being diagnosed at age 16, and the other two were diagnosed at ages 15 years (with duration of 5 years) and 28 years (duration 4 years.) The peak CP and PI occurred between 120–180 minutes in these subjects. Having a greater PI during the MMTT appeared to be associated with a lower ΔCP in these subjects.

In the T1D Exchange subjects who were selected based on presence of measurable CP, PI was also measurable in all 37 of them. To determine whether a potentially physiologically relevant PI concentration exists in the CP-positive, T1D exchange subjects, we stratified subjects according to PI values less or greater than 6 pmol/L based on their post-meal values. Twenty of these T1D subjects had a non-fasting PI >6 pmol/L and greater absolute PI and CP concentrations than those with a non-fasting PI below 6 pmol/L; however, the PI:CP ratio did not differ between the two groups. Interestingly, as seen in our local T1D cohort, in the group with greater PI concentrations, ΔCP was significantly lower than in those with lower stimulated PI concentrations. This finding suggests PI may be “preferentially secreted” following a meal stimulus compared to CP in long standing T1D or that there are differences in beta cell processing in individuals with T1D resulting in variation in beta-cell secretory products. Our findings further support the notion that residual beta cell function in long standing T1D, albeit it with processing deficiencies, may no longer be unexpected.

While we did not find any individuals in our local cohort with PI alone, it is still unclear why PI may be secreted in some individuals without measurable CP [17]. Decreased levels of the prohormone convertase PCSK1 mRNA and silencing of the INS promoter, resulting in incomplete transcription of the INS gene, have been recently reported in T1D pancreata [16]. These data may support the rational notion of disrupted prohormone processing in T1D individuals, something that has also been suggested in type 2 diabetes [19]. Alternatively, dysfunctional beta cells may simply rapidly secrete an incompletely processed product, where there is beta cell stress to the extent that PI is released prematurely, prior to proper cleavage that normally yields insulin and CP [12]. The elevated non-fasting PI:CP ratios in the T1D Exchange cohort and local T1D cohort compared to the stimulated PI:CP ratio in the healthy control group, is also supportive of the presence of residual dysfunctional beta cells.

We acknowledge the relatively small sample size of our healthy control group and T1D group as a limitation of this pilot study. However, we performed both a 90 minute and a subsequent 240 minute MMTT study on each participant in the healthy cohort on separate days in order to corroborate our own data. Our data was very consistent on an intra-individual basis and provides new insights into normal beta-cell secretory patterns following a standard MMTT. The limited availability of phenotypic and clinical information as well as the non-fasting PI and CP measurements in the T1D Exchange cohort is an additional limitation. Whether insulin administration prior to MMTT may have reduced the absolute peak CP concentration in these subjects is unclear [20], but the same would have been expected to occur to their PI responses. To diminish the possible effect of such, we required that their samples be obtained more than 15 minutes after commencing the meal and we did not include individuals who were hypoglycemic at the time of sample collection. Unfortunately, given limited sample availability, we were not able to obtain access to all the post MMTT samples from the T1D Exchange cohort to better characterize their responses. While limited in number, the MMTT data from our local T1D population suggests that CP, and particularly PI, may not peak until 120 minutes or later following a standard MMTT. Therefore, MMTT studies with hormone values collected for only 90 minutes may underestimate the maximum secretory potential of beta cells, particularly when PI is included in the analysis. Based on our findings and the limitations of our study, we suggest future studies should determine in an unbiased manner the true prevalence of residual proinsulin release in long standing T1D and the longitudinal nature of this interesting aspect of beta cell function.

## Conclusion

In conclusion, based on CP and PI measurements in T1D individuals with long standing disease and measurable CP, we found evidence of stimulated PI release, despite not demonstrating a significant nutrient stimulated CP response. These data suggest that while some individuals with T1D have residual beta cell function, it is characterized by an inability to fully process proinsulin to CP and insulin.

## Supporting information

S1 Table MMTT PI data local cohort. (XLSX)

S2 Table MMTT CP data local cohort. (XLSX)

S3 Table T1D Exchange cohort (n = 37). (XLSX)
